# Epigenetic aging and blood based neurodegeneration markers in LASI-DAD

**DOI:** 10.1016/j.tjpad.2026.100595

**Published:** 2026-05-14

**Authors:** Jung Ki Kim, Thalida E. Arpawong, Bharat Thyagarajan, Jennifer A. Smith, Sithara Vivek, Scott Ratliff, Sharmistha Dey, Jinkook Lee, Eileen M. Crimmins

**Affiliations:** a Davis School of Gerontology, University of Southern California, Los Angeles, CA, USA; b Department of Laboratory Medicine and Pathology, University of Minnesota, Minneapolis, MN, USA; c Survey Research Center, Institute for Social Research, University of Michigan, Ann Arbor, MI, USA; d Department of Biophysics, All India Institute of Medical Sciences, New Delhi, India; e Department of Economics and Center for Economic and Social Research, University of Southern California, Los Angeles, CA, USA

**Keywords:** Epigenetic aging, DNA methylation, Neurodegeneration biomarkers, Dementia, Low- and middle-income countries

## Abstract

DNA methylation (DNAm)-based epigenetic clocks are emerging biomarkers of biological aging and have been linked to cognitive decline and dementia, but their relationship with blood-based neurodegenerative biomarkers remains understudied in low- and middle-income countries (LMIC). Using the Longitudinal Aging Study in India-Diagnostic Assessment of Dementia (LASI-DAD), we examined whether epigenetic aging was associated with levels and changes in neurodegenerative biomarkers among adults aged ≥60 years. Seven epigenetic clocks were derived from DNAm data and related to plasma levels of glial fibrillary acidic protein (GFAP), neurofilament light (NfL), phosphorylated tau 181 (pTau181), total tau, Amyloid-β (Aβ)42, Aβ40 and Aβ42/Aβ40 measured at two time points. Baseline accelerated epigenetic aging was associated with higher levels of neurodegenerative biomarkers, including pTau181, GFAP, and NfL, with more consistent associations with increases in GFAP and NfL for morbidity- and mortality-trained clocks. These findings support the utility of epigenetic clocks as scalable tools for identifying risk of neurodegeneration in LMIC settings.

## 1 Introduction

Dementia is a leading cause of disability and dependency in older adults worldwide, with an estimated 57 million people currently living with dementia and about 10 million new cases occur every year, with numbers projected to rise sharply in the coming decades [1]. More than 60% of those affected reside in low- and middle-income countries (LMICs), where population aging is rapid but diagnostic resources remain limited [1]. Identifying scalable, blood-based markers that capture early neurodegenerative processes and upstream biological aging is therefore a critical priority for dementia research and prevention in these settings [2].

Blood biomarkers of neurodegeneration and Alzheimer’s disease-related dementias (ADRD) pathology have advanced rapidly in recent years and are increasingly recognized as indicators of risk for cognitive decline and dementia. Glial fibrillary acidic protein (GFAP) and neurofilament light chain (NfL) index astrocytic activation and axonal injury, respectively, neurodegeneration processes not specific to AD [[3], [4], [5]]. Plasma amyloid beta 42 to 40 ratio (Aβ42/Aβ40) captures amyloid deposition, while phosphorylated tau-181 (pTau181) reflects tau pathology closely linked to amyloid pathology. Total tau serves as a general marker of neuronal injury [6]. Together, these biomarkers are increasingly used to track neurodegenerative processes, cognitive decline, and dementia risk, and can be measured from a simple blood draw, making them efficient and cost-effective for large population-based studies, including in LMICs [2].

DNA methylation (DNAm)-based “epigenetic clocks” have been developed as molecular indicators of the overall biological aging and predictors of mortality and other aging outcomes [[7], [8], [9], [10]]. Second- and third-generation clocks, including PhenoAge [11], GrimAge2 [12], DunedinPACE [13], SystemsAge [14], and PhysAge [15], incorporate clinical risk factors, mortality-linked methylation patterns, and multi-system physiology measures, and better capture aging-related morbidity, functional decline, and mortality [8,10] than first-generation clocks such as Horvath and Hannum [16,17]. Accelerated epigenetic age has been observed in AD and linked to dementia and cognitive decline, suggesting that epigenetic clocks reflect biological processes contributing to neurodegeneration [18].

A small but emerging literature has begun to connect epigenetic clocks directly to blood-based neurodegenerative biomarkers. Cross-sectional and longitudinal studies show that epigenetic age acceleration is associated with higher NfL and amyloid markers, while faster aging measures predict increases in pTau, NfL, and GFAP over time, with some sex-specific effects [19,20]. However, this evidence comes largely from high-income or relatively homogeneous populations (e.g., Hispanic adults and predominantly European-ancestry women).

To address these gaps, we leveraged a population-based cohort of older Indian adults with repeated measures of epigenetic clocks and neurodegenerative biomarkers. India is home to one of the world’s largest and fastest-growing aging populations, yet remains underrepresented in aging and dementia research [21]. Using data from the Longitudinal Aging Study in India-Diagnostic Assessment of Dementia (LASI-DAD), we examine how accelerated epigenetic aging relates to levels and longitudinal change in blood-based markers of neurodegeneration and AD pathology. Recent analysis in LASI-DAD and a parallel U.S. cohort has shown that the neurodegenerative biomarkers such as GFAP and NfL are consistently associated with cognitive status and dementia in both settings, but that their ability to predict subsequent cognitive decline differs across countries [3]. Whether epigenetic clocks account for individual differences in these biomarkers or their changes over time in the Indian context remains an open question.

Focusing on a panel of first-, second-, and third-generation epigenetic clocks (Horvath, Hannum, PhenoAge, GrimAge2, SystemsAge, PhysAge, and DunedinPACE) and neurodegenerative biomarkers (GFAP, NfL, pTau181, total tau, Aβ42, Aβ40, and the Aβ42/Aβ40 ratio), we test four related questions: (1) whether baseline accelerated epigenetic aging is associated with baseline levels of neurodegenerative biomarkers; (2) whether baseline accelerated epigenetic aging is associated with subsequent levels of neurodegenerative biomarkers; (3) whether baseline accelerated aging predicts change in these biomarkers between waves; and (4) whether change in accelerated epigenetic aging over time is associated with concurrent change in neurodegenerative biomarkers. By addressing these questions in a large, population-based sample of older adults in India, we aim to clarify the extent to which epigenetic aging links to early markers of neurodegeneration in an LMIC setting.

## 2 Methods

### 2.1 Data and study population

We used data from LASI-DAD, an in-depth study of late-life cognition and dementia nested within the nationally representative LASI cohort of adults aged 60 years and older. LASI-DAD includes detailed neuropsychological assessments, informant interviews, and venous blood collection at two time points, hereafter referred to as wave 1 (2017–2019) and wave 2 (2022–2024) [21]. The blood collection enables repeated measurement of DNAm-based epigenetic clocks and plasma neurodegenerative biomarkers for sample individuals. This design allows us to examine association between baseline epigenetic age acceleration and subsequent biomarker levels, as well as within-person change over time in epigenetic aging and neurodegenerative biomarkers. LASI-DAD provides a unique opportunity to examine these associations in a population-based, socioeconomically diverse sample, and to expand the global relevance of research linking epigenetic aging to neurodegeneration.

Analyses were restricted to participants with DNAm-derived epigenetic clock measures and plasma neurodegenerative biomarkers at both waves, and complete data on age, sex, education, caste, residency, smoking, vascular and metabolic factors, and depression. DNA methylation and plasma biomarkers were derived from LASI-DAD participants who consented to venous blood collection and had sufficient sample volume and assay quality [3]. Of 1409 participants with epigenetic clock data at Wave 1, 875 also had epigenetic measures at Wave 2; 177 lacked neurodegenerative biomarkers and 123 had missing covariates, yielding a final analytic sample of 575 (Supplementary Table 1). Although multilevel approaches can better handle missing data, we restricted analyses to participants with complete biomarker and DNAm data at both waves for consistency. This restriction may affect generalizability if missingness is not random, although differences between included and excluded participants were modest. Participants with epigenetic data at both waves were younger (67.9 vs. 72.7 years, p < 0.001) and more often female (51.2% vs. 43.9%, p = 0.008) than those with only Wave 1 data; those with and without missing covariates did not differ in mean age or in the proportion female. The time interval (in years) between waves was used to annualize change measures. The sample ranges from 437 to 560 depending on missing neurodegenerative biomarkers. Sample characteristics are presented in Table 1.

**Table 1:** Sample Description (LASI-DAD).

	N	Mean (SD) / %
Mean Age	575	68.00 (6.00)
%Female	575	51.5%
%Education	575	
No formal education		49.8%
1–8 years of schooling		31.4%
≥9 years of schooling		18.8%
%Caste^a^	575	
Scheduled caste/tribe		23.4%
Other backward class		46.5%
No caste or other caste		30.1%
%Rural residency	575	74.0%
%Smoking status	575	
Never smoker		80.8%
Former smoker		4.7%
Current smoker		14.5%
%High blood pressure (SBP>140 mmHg or DBP>90 mmHg)	575	43.2%
%High HbA1c (≥6.5%)	575	21.7%
Mean total to HDL cholesterol ratio	575	4.25 (1.17)
%Overweight (≥25kg/m^2^)	575	27.1%
Mean eGFR (mL/min/1.73m^2^)^b^	575	74.56 (21.18)
%Depression^c^	575	8.1%
Epigenetic Clocks		
Wave 1		
Horvath	575	75.52 (7.87)
Hannum	575	50.86 (7.01)
PhenoAge	575	64.09 (8.32)
GrimAge2	575	72.49 (5.98)
PhysAge	575	65.97 (8.93)
SystemsAge	575	72.94 (12.69)
DunedinPACE	575	1.21 (0.11)
Wave 2		
Horvath	575	80.27 (8.27)
Hannum	575	54.56 (7.01)
PhenoAge	575	68.85 (8.65)
GrimAge2	575	76.01 (6.12)
PhysAge	575	67.99 (9.65)
SystemsAge	575	76.48 (13.64)
DunedinPACE	575	1.23 (0.11)
Neurodegenerative Markers		
Wave 1		
GFAP	548	132.42 (93.11)
NfL	535	33.40 (24.31)
PTau181	556	45.34 (44.42)
Total tau	560	3.61 (7.23)
Aβ42/40 ratio	437	0.06 (0.03)
Aβ42	438	3.54 (2.18)
Aβ40	524	55.01 (41.17)
Wave 2		
GFAP	548	126.67 (68.61)
NfL	535	30.04 (21.20)
pTau181	556	43.98 (29.36)
Total tau	560	1.58 (1.34)
Aβ42/40 ratio	437	0.06 (0.02)
Aβ42	438	4.32 (2.19)
Aβ40	524	65.49 (28.19)

a Caste categories were defined as scheduled caste/scheduled tribe (constitutionally recognized groups, generally with the lowest social status), other backward class (government-defined socially and educationally disadvantaged groups, with intermediate social status), and no caste/other caste (generally highest social status; reference group).

b eGFR was calculated using a CKD-EPI equation based on serum creatinine, cystatin C, age and sex, logged due to skewed values.

c The CIDI-SF (Composite International Diagnostic Interview Short Form) for Major Depressive Episode (3+ out of 7).

### 2.2 Measures

#### 2.2.1 Neurodegenerative biomarkers

At both waves, venous blood samples were collected and assayed for a panel of plasma biomarkers related to AD and neurodegeneration, using a Quanterix HD-X Simoa immunoassay platform. More details on assays and validation procedures are provided elsewhere [3]. These included Aβ40, Aβ42, GFAP, NfL, pTau181, and total tau. Aβ42/40 ratio was calculated by dividing Aβ42 by Aβ40. Because the distributions of GFAP, NfL, pTau181, and total tau were right skewed, we applied a natural log transformation before analysis. The Aβ42/40 ratio was analyzed using its original scale. Descriptive statistics are presented in Table 1 and Supplementary Table 1. For each biomarker, change was calculated as the difference between the levels at Waves 1 and 2 divided by follow-up time (years), yielding an annualized change. For log-transformed biomarkers, this reflects annualized change in log-biomarker; for Aβ42, Aβ40, and their ratio, it reflects annualized change in the raw values.

#### 2.2.2 Epigenetic clocks

DNAm data were available at both waves, and multiple epigenetic clocks were computed using established algorithms, including first-generation (Horvath, Hannum), second-generation (PhenoAge, GrimAge2, PhysAge, SystemsAge), and third-generation (DunedinPACE) measures. These clocks are based on DNA methylation levels at selected genomic sites called CpG (Cytosine-phosphate-Guanine) and capture different aspects of biological aging, including mortality risk and multisystem physiological decline. The detailed procedures for DNAm processing and epigenetic clock construction are described in Supplementary material. While prior studies highlight GrimAge, PhenoAge, and DunedinPACE as leading predictors of aging-related outcomes, evidence for other clocks in diverse populations, particularly in LMIC, is limited. Therefore, we included all seven clocks to both replicate prior findings and examine differences in associations with neurodegenerative biomarkers in this understudied Indian cohort. Our approach was exploratory and not pre-registered; all clocks were analyzed without a priori selection, using the full LASI-DAD sample with available data to maximize statistical power, while acknowledging that the study was not designed to test specific clocks.

We employed epigenetic age acceleration measures, defined as the residuals from regressions of epigenetic age on chronological age which is interpreted as faster or slower epigenetic aging at each wave. For DunedinPACE, which is scaled as a rate of aging (values greater than 1 indicating a faster pace than chronological aging), we used the raw DunedinPACE values at each wave. For Horvath, Hannum, PhenoAge, GrimAge2, PhysAge, and SystemsAge, we defined annualized change in epigenetic age acceleration as the difference between Wave 2 and Wave 1 acceleration values divided by follow-up time in years; for DunedinPACE, we used the change in the raw measure per year.

#### 2.2.3 Covariates

Covariates included chronological age at Wave 1 (years), sex, education (no formal education (reference), 1–8 years, and ≥9 years), caste (no caste/other caste (reference), scheduled caste/tribe, and other backward class, see Table 1 for details), rural residency, smoking status (never (reference), former, and current), and additional vascular and metabolic factors such as high blood pressure, high HbA1c, total to HDL cholesterol ratio, overweight, kidney function (eGFR), and depression. This adjustment set was selected to capture key sociodemographic, behavioral and biological risk factors that could confound associations between epigenetic aging and neurodegenerative biomarkers.

##### 2.2.3.1 Analysis

To facilitate comparability and interpretation, all epigenetic clock measures and neurodegenrative biomarkers were standardized. We z-scored baseline epigenetic age acceleration for each age-based clock at Wave 1, DunedinPACE at Wave 1, and annualized change in each clock. For biomarkers, we z-scored log-transformed levels of GFAP, NfL, p-tau181, and total tau at each wave, the Aβ42/40 ratio at each wave, and annualized change for all biomarkers. Regression coefficients, therefore, represent the standard deviation (SD) difference in biomarker outcomes per one SD difference in epigenetic clock acceleration, conditional on covariates.

We estimated four sets of linear regression models corresponding to our research questions. Model A examined whether baseline accelerated epigenetic aging was associated with baseline biomarker levels. Model B examined whether baseline accelerated epigenetic age acceleration was associated with subsequent biomarker levels. Model C examined whether baseline accelerated epigenetic aging was associated with change in biomarkers. Model D evaluated whether change in accelerated epigenetic aging was associated with concurrent biomarker change. All four models adjusted for the same covariates (age, sex, education, rural residency, caste, smoking status, high blood pressure, high HbA1c, total to HDL cholesterol ratio, overweight, kidney function (eGFR), and depression), with additional adjustment of baseline biomarker levels for Models C and D.

For each model, we ran separate linear regressions for all combinations of epigenetic clocks (Horvath, Hannum, PhenoAge, GrimAge2, PhysAge, SystemsAge, and DunedinPACE) and neurodegenerative biomarkers (GFAP, NfL, pTau181, total tau, Aβ42, Aβ40, and Aβ42/40). Analyses applied Wave 1 biomarker weights to account for sampling design and differential non-response.

To address multiple testing, we used the Benjamini–Hochberg false discovery rate (FDR) procedure within each model set, reporting both nominal and FDR-adjusted p values. Associations with FDR<0.05 were considered statistically significant, while those with nominal p < 0.05 but FDR≥0.05 were treated as suggestive. All analyses were conducted in RStudio 2026.01 (R Foundation for Statistical Computing).

## 3 Results

### 3.1 Baseline epigenetic age acceleration and baseline neurodegenerative biomarkers

**Fig. 1:**
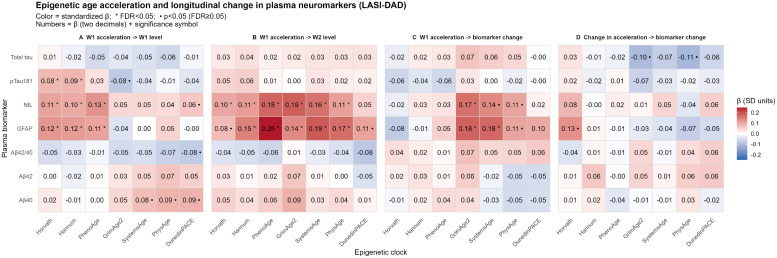
Associations Between Epigenetic Age Acceleration and Plasma Neurodegenerative Biomarkers across Two Waves of LASI-DAD. Numbers=standardized β + significance symbol *FDR<0.05; ^●^p < 0.05 (FDR≥0.05) P value and FDR are available in supplementary Tables 2–5. Age, sex, education, caste, rural residency, smoking, high blood pressure, high HbA1c, total to HDL cholesterol ratio, overweight, logged eGFR and depression are controlled in each model.

We examined cross-sectional associations between epigenetic age acceleration and neurodegenerative biomarker levels at baseline (Fig. 1A, Supplementary Table 2). Accelerated epigenetic aging was consistently associated with higher plasma GFAP and NfL, particularly for first-generation clocks (Horvath, Hannum) and PhenoAge. The largest effects were observed for PhenoAge with NfL (β=0.125, p < 0.001, FDR=0.002) and Horvath with GFAP (β=0.122, p < 0.001, FDR=0.002), with similar estimates for Hannum. DunedinPACE showed a modest association with NfL that did not survive multiple testing correction, while GrimAge2, SystemsAge, and PhysAge were not significantly associated with these markers.

Associations with tau- and amyloid-related biomarkers were weaker and less consistent. PTau181 was positively associated with Horvath and Hannum (β≈0.08, p < 0.01, FDR=0.034), but not with other clocks. No significant associations were found for total tau or Aβ42/40 after correction. Modest positive associations with Aβ40 were observed for SystemsAge, PhysAge, and DunedinPACE, though only PhysAge and DunedinPACE remained marginally significant after correction. Overall, epigenetic age acceleration was more strongly associated with markers of neurodegeneration and astrocytic activation than with amyloid or tau pathology.

### 3.2 Baseline epigenetic age acceleration and subsequent neurodegenerative biomarkers

We next examined whether baseline epigenetic age acceleration was associated with biomarker levels at follow-up (Wave 2) (Fig. 1B, Supplementary Table 3). Accelerated epigenetic aging at Wave 1 was consistently associated with higher GFAP and NfL at Wave 2 across multiple clocks. The largest effects were observed for PhenoAge (GFAP: β=0.248, p < 0.001, FDR<0.001; NfL: β=0.178, p < 0.001, FDR<0.001), followed by SystemsAge (GFAP: β=0.189, p < 0.001, FDR<0.001; NfL: β=0.162, p < 0.001, FDR<0.001) and GrimAge2 (GFAP: β=0.136, p = 0.005, FDR=0.027; NfL: β=0.185, p < 0.001, FDR<0.001). Hannum and PhysAge also showed significant associations, while DunedinPACE showed weaker, non-significant associations with NfL and only a marginal association with GFAP that did not survive multiple testing correction. No significant associations were detected with pTau181, total tau, or amyloid-related biomarkers after correction.

### 3.3 Baseline epigenetic age acceleration and change in neurodegenerative biomarkers

We then evaluated whether baseline epigenetic age acceleration was associated with annualized change in neurodegenerative biomarkers (Fig. 1C, Supplementary Table 4). GrimAge2 showed significant associations with increases in both GFAP and NfL over time (GFAP: β=0.181, *p* = 0.002, FDR=0.037; NfL: β=0.166, *p* = 0.002, FDR=0.040). SystemsAge was also associated with steeper increases in GFAP (β=0.175, *p* < 0.001, FDR=0.029) and showed a suggestive association with NfL (β=0.136, p = 0.006, FDR=0.071). PhysAge demonstrated nominal associations with both GFAP and NfL, although these did not survive multiple testing correction. No other clocks were significantly associated with changes in these biomarkers. No significant associations were observed between baseline epigenetic aging and changes in tau or amyloid-related biomarkers after adjustment for sociodemographic, vascular, and metabolic factors.

### 3.4 Change in epigenetic aging and change in neurodegenerative biomarkers

Associations between changes in epigenetic age acceleration and changes in neurodegenerative biomarkers were generally weak and did not survive correction for multiple testing (Fig. 1D, Supplementary Table 5). While a nominal positive association was observed between change in Horvath age acceleration and change in GFAP, and inverse associations were observed for GrimAge2 and PhysAge with total tau, these findings were not robust after FDR adjustment. Inverse associations were observed for GrimAge2 and PhysAge with total tau but effect sizes were small and did not survive FDR correction. No consistent associations were detected for NfL, pTau181, or amyloid-related biomarkers, suggesting that changes in 4–5 year interval in epigenetic aging may not closely track concurrent changes in neurodegenerative processes.

## 4 Discussion

This study provides evidence that DNAm-based epigenetic clocks are associated with blood-based neurodegenerative biomarkers in older Indian adults, with stronger and more consistent longitudinal associations observed for second- and third-generation measures. Using longitudinal LASI-DAD data, we found that accelerated epigenetic aging was associated with higher follow-up levels and steeper increases in GFAP and NfL, particularly for clocks capturing multisystem physiological dysregulation and mortality risk (PhenoAge, GrimAge2, SystemsAge, and PhysAge). The stronger longitudinal associations observed for second- and third-generation clocks likely reflect their design, as they are explicitly trained on phenotypic indicators of disease and mortality rather than chronological age alone. This makes them more biologically informative and relevant to disease pathways, capturing systemic aging processes linked to brain aging and neuroinflammation, potentially through vascular, metabolic, or immunological mechanisms underlying neurodegeneration.

On the other hand, DunedinPACE, which indexes the rate of biological aging rather than cumulative methylation, showed weaker and less consistent associations with neurodegenerative processes, suggesting only partial overlap. In contrast, first-generation clocks were more pronounced for cross-sectional associations. This may reflect greater heterogeneity in age-related exposures and differences in how phenotypic risk is structured in this LMIC setting, such that first-generation clocks—more closely aligned with chronological age and general aging processes—better capture baseline variation, whereas second- and third-generation measures better capture longitudinal change.

Our findings align with evidence from high-income, predominantly European-ancestry populations, which report associations between epigenetic age acceleration and markers such as GFAP, NfL, and amyloid-related measures [[8], [9], [10],^19^,^20^]. Particularly, our results show stronger and more consistent associations with markers of neurodegeneration than with amyloid or tau pathology. By demonstrating similar patterns in an Indian population, our study extends this evidence to a more diverse context and supports the broader applicability of epigenetic aging measures across populations, addressing an important gap in the epigenetic aging literature in diverse populations. Particularly in LMICs where imaging and CSF-based diagnostics are often unavailable, blood-based aging measures offer a promising avenue for scalable risk stratification. If epigenetic clocks can identify individuals at heightened risk of subclinical neurodegeneration before cognitive decline is clinically apparent, they may help fill a critical gap in dementia prevention strategies in under-resourced settings.

The consistent associations between epigenetic age acceleration and higher levels of GFAP and NfL both at follow-up and over time support the hypothesis that accelerated epigenetic aging may predispose individuals to early neurodegenerative deterioration. GFAP, a marker of reactive astrogliosis, reflects glial activation and neuroinflammatory responses, while NfL reflects axonal degeneration and injury to large-caliber myelinated fibers. These proteins are widely recognized as sensitive and dynamic indicators of subclinical neurodegeneration, with elevations seen years before the onset of clinical symptoms. Our findings suggest that faster epigenetic aging may be associated with or parallel these early pathophysiological changes.

In contrast to GFAP and NfL, associations between epigenetic clocks and tau- or amyloid-related biomarkers were fewer and less consistent. Baseline epigenetic aging showed only weak associations with pTau181 and Aβ42/40 ratio, which did not survive multiple testing correction. This pattern is consistent with prior studies reporting stronger links between epigenetic aging and markers of neuroinflammation or neurodegeneration than with amyloid or tau pathology [19,20]. GFAP and NfL are more responsive to ongoing neural injury and inflammation, whereas amyloid and tau changes often occur earlier and plateau before clinical symptoms. As a result, the window during which epigenetic aging and tau/amyloid markers co-vary may be narrower and not fully captured within our follow-up period. Neurodegenerative biomarkers evolve gradually, making the timing of measurements critical for detecting change. The 4.5–5-year interval in our study aligns with timeframes over which meaningful changes in blood-based biomarkers may be observed, particularly in preclinical and early neurodegeneration. This is especially relevant in older populations, where neurodegenerative processes accelerate, increasing the likelihood of detectable within-person change. Prior studies show that GFAP, NfL, and pTau change gradually over multi-year periods rather than short-term intervals [22,23]. At the same time, baseline differences observed in our study suggest that some divergence in these biomarkers may have occurred before initial assessment, indicating that our findings likely reflect ongoing progression rather than onset of neurodegenerative processes.

Observed effect sizes, presented in standard deviation units, were modest. This is expected in a community-dwelling, largely non-demented population where variability in neurodegenerative biomarkers may be limited and pathological processes are likely at earlier stages. In such settings, even small differences may reflect meaningful shifts in underlying biological aging trajectories. Moreover, the consistent associations observed across multiple clocks and biomarkers, particularly for GFAP and NfL, support the biological relevance of these findings despite their modest magnitude. From a clinical perspective, small shifts in these biomarkers at the population level may have important implications for early risk stratification and the identification of individuals at increased risk for future neurodegenerative change.

Socioeconomic and environmental factors specific to the Indian context should be considered when interpreting these findings. Substantial heterogeneity in early-life conditions, education, nutrition, and environmental stressors can influence both epigenetic aging and neurodegenerative risk. For example, greater exposure to infectious burden, inadequate diet, air pollution, and socioeconomic adversity may accelerate biological aging, potentially strengthening or modifying associations between epigenetic clocks and neurodegenerative biomarkers. Conversely, differences in lifestyle, social structure, and survival patterns may limit comparability with high-income populations. Thus, while our findings support the relevance of epigenetic aging measures in an LMIC setting, caution is needed when extrapolating effect sizes or mechanisms across populations with differing environmental and socioeconomic contexts.

Some limitations are noted. The follow-up period, while sufficient to detect intermediate changes in neurodegenerative biomarkers, may be short to capture longer-term trajectories. Due to limited availability in the analytic sample, genetic risk factors such as APOE genotype, which could modify the relationship between epigenetic aging and neurodegeneration, were not included. Measuring change using annualized difference scores, though interpretable and common, can be sensitive to measurement error and regression to the mean. Moreover, changes in epigenetic age acceleration are complex to interpret biologically and may reflect both true biological change and technical noise. Future studies should explore longer-term trajectories and link them to clinical outcomes such as mild cognitive impairment or dementia onset.

Nonetheless, this study strengthens evidence that epigenetic aging captures biologically meaningful variation linked to neurodegenerative processes, demonstrating its relevance in a large, underrepresented LMIC population. By extending prior findings beyond European-ancestry, high-income settings, our results underscore the importance of including diverse populations in biomarker research to ensure global validity and equity. Notably, accelerated epigenetic aging was consistently associated with markers of astrocytic activation and axonal injury, highlighting its potential as a scalable, blood-based tool for early detection, risk stratification, and dementia prevention. Future research should clarify the biological pathways connecting methylation signatures to neurodegeneration, leverage multi-omic and imaging data, and refine clock calibration in diverse populations to maintain cultural and biological relevance. Overall, these findings position epigenetic clocks as promising molecular indicators that can bridge aging biology and population health, offering actionable insights for promoting cognitive health worldwide.

## Funding

BrightFocus Bold Ideas Initiatives Program CA2023001

National Institute on Aging, National Institutes of Health R01AG051125, R01AG088003

## Declaration of generative AI and ai-assisted technologies in the writing process

During the preparation of this manuscript, OpenAI’s ChatGPT was used to refine the readability of a few selected sentences of the text. The authors take full responsibility for the manuscript.

## Data statement

Neurodegenerative biomarker data are available through the Gateway to Global Aging Data Enclave (https://g2aging.org/enclave) and the Alzheimer’s Disease Data Initiative (ADDI) AD Workbench (https://www.alzheimersdata.org/ad-workbench). Researchers can get access to the data by completing the required data use agreement. Epigenetic data will be available through the National Institute on Aging Genetics of Alzheimer’s Disease Data Storage Site (NIAGADS).

## CRediT authorship contribution statement

**Jung Ki Kim:** Writing – review & editing, Writing – original draft, Visualization, Methodology, Investigation, Formal analysis, Data curation, Conceptualization. **Thalida E. Arpawong:** Writing – review & editing, Data curation. **Bharat Thyagarajan:** Supervision, Project administration, Data curation. **Jennifer A. Smith:** Writing – review & editing, Supervision, Project administration, Data curation. **Sithara Vivek:** Writing – review & editing, Data curation. **Scott Ratliff:** Data curation. **Sharmistha Dey:** Funding acquisition. **Jinkook Lee:** Writing – review & editing, Supervision, Project administration, Funding acquisition. **Eileen M. Crimmins:** Writing – review & editing, Supervision, Project administration, Data curation, Conceptualization.

## Declaration of competing interest

The authors declare that they have no known competing financial interests or personal relationships that could have appeared to influence the work reported in this paper.

## Appendix Supplementary materials

Image, application 1
