# Supplementary material for: Epigenetic aging and blood based neurodegeneration markers in LASI-DAD
Source: J Prev Alzheimers Dis. 2026 May 14;13(7):100595. doi: 10.1016/j.tjpad.2026.100595 (PMC13196560; doi:10.1016/j.tjpad.2026.100595)
Supplement: Supplementary file 1 [file mmc1.docx]

Supplementary Material

DNAm Processing and Epigenetic Clock Construction

DNA methylation (DNAm) was assayed using the Illumina Infinium MethylationEPIC v2.0 BeadChip. Standard preprocessing and quality control procedures were applied, including sample- and probe-level filtering. Specifically, samples with poor quality metrics, sex mismatches, low SNP–probe correlations, and duplicates were excluded, resulting in 2,290 high-quality samples, including 1,409 participants at wave 1 and 881 at wave 2, with 875 having data at both waves. At the probe level, probes with detection p-values >0.01 in more than 5% of samples were removed, replicate probes were excluded, and low bead-count measurements were set to missing, yielding approximately 928,000 CpG sites for analysis. To minimize technical variation, longitudinal samples from the same individual were processed on the same plate and assayed together, and preprocessing procedures were harmonized with those used in the US Health and Retirement Study. While the EPIC v2.0 array differs from earlier platforms (e.g., 450K and EPIC v1.0) used to develop many epigenetic clocks, the majority of CpG sites were successfully captured. The proportion of missing CpGs varied by clock, ranging from minimal for PhysAge (0.2%) and PhenoAge (3.5%) to higher for GrimAge2 (18%) and DunedinPACE (16.8%), with missingness primarily due to probe absence rather than quality failure. Clock-specific procedures were used to address missing CpGs, including imputation following developer recommendations where appropriate, ensuring consistency with established implementations. Nevertheless, minor differences in CpG coverage across platforms may contribute to some variability in clock estimates. While direct cross-platform validation analyses were not feasible within the dataset, our harmonized preprocessing pipeline and adherence to published clock construction protocols assure the robustness of the derived clock measures.

Supplementary Table 1. Distribution of Neurodegenerative Biomarkers

|  | Median | IQR |
| --- | --- | --- |
| Wave 1 |  |  |
| GFAP | 114.30 | 89.75 |
| NfL | 27.25 | 19.05 |
| pTau181 | 36.58 | 24.71 |
| Total tau | 1.96 | 1.85 |
| Aβ42/40 ratio | 0.06 | 0.03 |
| Aβ42 | 3.48 | 3.10 |
| Aβ40 | 51.05 | 59.37 |
| Wave 2 |  |  |
| GFAP | 109.84 | 73.40 |
| NfL | 23.19 | 16.76 |
| pTau181 | 38.12 | 21.45 |
| Total tau | 1.22 | 1.33 |
| Aβ42/40 ratio | 0.06 | 0.02 |
| Aβ42 | 4.25 | 2.88 |
| Aβ40 | 64.03 | 34.56 |

IQR: Interquartile Range

Supplementary Table 2: Associations Between Baseline Epigenetic Age Acceleration and Wave 1 Plasma Neurodegenerative Biomarkers in LASI-DAD

| Neuro-markers | Horvath1 | Hannum | PhenoAge | GrimAge2 | SystemsAge | PhysAge | DunedinPACE |
| --- | --- | --- | --- | --- | --- | --- | --- |
| Total Tau | 0.012 (0.032), p=0.704, FDR=0.821 | -0.020 (0.034), p=0.552, FDR=0.694 | -0.055 (0.033), p=0.098, FDR=0.267 | -0.036 (0.039), p=0.354, FDR=0.510 | -0.046 (0.034), p=0.176, FDR=0.363 | -0.061 (0.034), p=0.072, FDR=0.227 | -0.014 (0.034), p=0.689, FDR=0.821 |
| PTau181 | **0.082 (0.029), p=0.006, FDR=0.034** | **0.086 (0.030), p=0.005, FDR=0.034** | 0.034 (0.030), p=0.255, FDR=0.391 | -0.082 (0.035), p=0.021, FDR=0.093 | -0.040 (0.031), p=0.196, FDR=0.363 | -0.012 (0.031), p=0.704, FDR=0.821 | -0.039 (0.031), p=0.219, FDR=0.363 |
| NfL | **0.107 (0.030), p<0.001, FDR=0.004** | **0.098 (0.031), p=0.002, FDR=0.013** | **0.125 (0.031), p<0.001, FDR=0.002** | 0.045 (0.036), p=0.211, FDR=0.363 | 0.045 (0.032), p=0.160, FDR=0.363 | 0.042 (0.032), p=0.190, FDR=0.363 | 0.064 (0.032), p=0.044, FDR=0.156 |
| GFAP | **0.122 (0.030), p<0.001, FDR=0.002** | **0.116 (0.031), p<0.001, FDR=0.004** | **0.107 (0.031), p<0.001, FDR=0.006** | -0.042 (0.037), p=0.255, FDR=0.391 | 0.002 (0.032), p=0.939, FDR=0.954 | 0.051 (0.032), p=0.113, FDR=0.291 | -0.004 (0.033), p=0.913, FDR=0.954 |
| Aβ42/40 ratio | -0.051 (0.037), p=0.166, FDR=0.363 | -0.034 (0.039), p=0.378, FDR=0.529 | -0.013 (0.038), p=0.736, FDR=0.839 | -0.054 (0.044), p=0.222, FDR=0.363 | -0.049 (0.039), p=0.215, FDR=0.363 | -0.069 (0.039), p=0.079, FDR=0.227 | -0.078 (0.039), p=0.045, FDR=0.156 |
| Aβ42 | 0.002 (0.036), p=0.948, FDR=0.954 | -0.023 (0.038), p=0.539, FDR=0.694 | 0.006 (0.037), p=0.872, FDR=0.950 | 0.029 (0.044), p=0.504, FDR=0.668 | 0.050 (0.038), p=0.199, FDR=0.363 | 0.068 (0.039), p=0.079, FDR=0.227 | 0.054 (0.038), p=0.154, FDR=0.363 |
| Aβ40 | 0.023 (0.034), p=0.498, FDR=0.668 | -0.006 (0.036), p=0.858, FDR=0.950 | 0.002 (0.035), p=0.954, FDR=0.954 | 0.045 (0.041), p=0.276, FDR=0.410 | 0.076 (0.037), p=0.039, FDR=0.156 | 0.087 (0.036), p=0.018, FDR=0.086 | 0.089 (0.037), p=0.016, FDR=0.086 |

Note: Neuro markers logged and z scored. All models adjusted for age, sex, education, rural residency, caste, smoking status, high blood pressure, high HbA1c, total to HDL cholesterol ratio, overweight, logged eGFR and depression

| Supplementary Table 3: Associations Between Baseline Epigenetic Age Acceleration and Wave 2 Plasma Neurodegenerative Biomarkers in LASI-DAD |
| --- |

| Neuro-markers | Horvath1 | Hannum | PhenoAge | GrimAge2 | SystemsAge | PhysAge | DunedinPACE |
| --- | --- | --- | --- | --- | --- | --- | --- |
| Total Tau | 0.025 (0.042), p=0.555, FDR=0.755 | 0.036 (0.041), p=0.388, FDR=0.656 | 0.017 (0.045), p=0.714, FDR=0.833 | 0.024 (0.053), p=0.654, FDR=0.821 | 0.030 (0.047), p=0.527, FDR=0.755 | 0.032 (0.046), p=0.484, FDR=0.74 | 0.033 (0.046), p=0.470, FDR=0.74 |
| PTau181 | 0.049 (0.038), p=0.192, FDR=0.496 | 0.056 (0.037), p=0.132, FDR=0.403 | 0.010 (0.041), p=0.808, FDR=0.900 | 0.001 (0.047), p=0.989, FDR=0.989 | 0.031 (0.042), p=0.465, FDR=0.740 | 0.019 (0.041), p=0.643, FDR=0.821 | 0.017 (0.041), p=0.681, FDR=0.833 |
| NfL | **0.102 (0.037), p=0.001, FDR=0.027** | **0.114 (0.037), p=0.002, FDR=0.015** | **0.178 (0.040), p<0.001, FDR<0.001** | **0.185 (0.046), p<0.001, FDR<0.001** | **0.162 (0.041), p<0.001, FDR<0.001** | **0.110 (0.041), p=0.007 FDR=0.031** | 0.052 (0.041), p=0.211, FDR=0.517 |
| GFAP | 0.077 (0.039), p=0.047, FDR=0.175 | **0.146 (0.039), p<0.001, FDR=0.001** | **0.248 (0.041), p<0.001, FDR<0.001** | **0.136 (0.048), p=0.005, FDR=0.027** | **0.189 (0.043), p<0.001, FDR<0.001** | **0.168 (0.042), p<0.001, FDR=0.001** | 0.106 (0.043), p=0.014, FDR=0.056 |
| Aβ42/40 ratio | -0.043 (0.045), p=0.345, FDR=0.642 | -0.050 (0.047), p=0.283, FDR=0.603 | -0.062 (0.050), p=0.223, FDR=0.521 | 0.006 (0.059), p=0.921, FDR=0.960 | -0.028 (0.052), p=0.587, FDR=0.777 | -0.045 (0.052), p=0.386, FDR=0.656 | -0.085 (0.051), p=0.094, FDR=0.308 |
| Aβ42 | 0.026 (0.044), p=0.551, FDR=0.755 | 0.007 (0.046), p=0.886, FDR=0.944 | 0.018 (0.050), p=0.712, FDR=0.833 | 0.066 (0.058), p=0.255, FDR=0.568 | 0.009 (0.051), p=0.863, FDR=0.939 | 0.003 (0.051), p=0.960, FDR=0.980 | -0.046 (0.050), p=0.353, FDR=0.642 |
| Aβ40 | 0.053 (0.040), p=0.181, FDR=0.492 | 0.038 (0.041), p=0.352, FDR=0.642 | 0.059 (0.044), p=0.179, FDR=0.492 | 0.092 (0.051), p=0.0708, FDR=0.248 | 0.032 (0.046), p=0.498, FDR=0.740 | 0.042 (0.045), p=0.353, FDR=0.642 | 0.013 (0.045), p=0.780, FDR=0.888 |

Note: Neuro markers logged and z scored. All models adjusted for age, sex, education, rural residency, caste, smoking status, high blood pressure, high HbA1c, total to HDL cholesterol ratio, overweight, logged eGFR and depression

Supplementary Table 4: Associations Between Baseline Epigenetic Age Acceleration and Change in Plasma Neurodegenerative Biomarkers Between Waves in LASI-DAD

| Neuro-markers | Horvath1 | Hannum | PhenoAge | GrimAge2 | SystemsAge | PhysAge | DunedinPACE |
| --- | --- | --- | --- | --- | --- | --- | --- |
| Total Tau | -0.025 (0.043), p=0.561, FDR=0.819 | 0.021 (0.043), p=0.631, FDR=0.836 | 0.026 (0.047), p=0.585, FDR=0.819 | 0.068 (0.054), p=0.208, FDR=0.794 | 0.056 (0.048), p=0.245, FDR=0.794 | 0.046 (0.048), p=0.340, FDR=0.794 | -0.002 (0.048), p=0.962, FDR=0.965 |
| PTau181 | -0.062 (0.039), p=0.115, FDR=0.626 | -0.039 (0.039), p=0.314, FDR=0.794 | -0.060 (0.043), p=0.163, FDR=0.794 | 0.026 (0.050), p=0.603, FDR=0.821 | 0.002 (0.044), p=0.965, FDR=0.965 | -0.024 (0.044), p=0.580, FDR=0.819 | -0.004 (0.044), p=0.934, FDR=0.965 |
| NfL | -0.025 (0.043), p=0.567, FDR=0.819 | 0.027 (0.045), p=0.544, FDR=0.819 | 0.032 (0.048), p=0.503, FDR=0.819 | **0.166 (0.055), p=0.002, FDR=0.040** | 0.136 (0.049), p=0.006, FDR=0.071 | 0.105 (0.048), p=0.029, FDR=0.286 | 0.015 (0.049), p=0.763, FDR=0.881 |
| GFAP | -0.080 (0.045), p=0.078, FDR=0.480 | -0.013 (0.046), p=0.774, FDR=0.881 | 0.053 (0.050), p=0.292, FDR=0.794 | **0.181 (0.057), p=0.002, FDR=0.037** | **0.175 (0.051), p<0.001, FDR=0.029** | 0.105 (0.050), p=0.037, FDR=0.302 | 0.099 (0.051), p=0.052, FDR=0.362 |
| Aβ42/40 ratio | 0.038 (0.048), p=0.435, FDR=0.819 | 0.021 (0.050), p=0.681, FDR=0.857 | 0.008 (0.054), p=0.885, FDR=0.942 | 0.072 (0.063), p=0.258, FDR=0.794 | 0.045 (0.056), p=0.421, FDR=0.819 | 0.048 (0.056), p=0.386, FDR=0.819 | 0.062 (0.054), p=0.253, FDR=0.794 |
| Aβ42 | 0.019 (0.048), p=0.699, FDR=0.857 | 0.033 (0.050), p=0.515, FDR=0.819 | 0.012 (0.054), p=0.820, FDR=0.893 | 0.057 (0.064), p=0.373, FDR=0.819 | -0.017 (0.056), p=0.765, FDR=0.881 | -0.055 (0.056), p=0.326, FDR=0.794 | -0.053 (0.054), p=0.333, FDR=0.794 |
| Aβ40 | -0.012 (0.045), p=0.798, FDR=0.888 | 0.019 (0.046), p=0.683, FDR=0.857 | 0.040 (0.050), p=0.420, FDR=0.819 | 0.038 (0.058), p=0.506, FDR=0.819 | -0.029 (0.053), p=0.579, FDR=0.819 | -0.055 (0.051), p=0.281, FDR=0.794 | -0.050 (0.051), p=0.322, FDR=0.794 |

Note: Neuro markers logged and z scored. All models adjusted for age, sex, education, rural residency, caste, smoking status, high blood pressure, high HbA1c, total to HDL cholesterol ratio, overweight, logged eGFR and depression, and for change models, the standardized baseline biomarker level at wave 1.

Supplementary Table 5: Associations Between Change in Epigenetic Age Acceleration and Concurrent Change in Plasma Neurodegenerative Biomarkers in LASI-DAD

| Neuro-markers | Horvath1 | Hannum | PhenoAge | GrimAge2 | SystemsAge | PhysAge | DunedinPACE |
| --- | --- | --- | --- | --- | --- | --- | --- |
| Total Tau | 0.032 (0.042), p=0.444, FDR=0.865 | -0.013 (0.042), p=0.757, FDR=0.944 | -0.021 (0.041), p=0.615, FDR=0.941 | -0.105 (0.040), p=0.009, FDR=0.202 | -0.074 (0.042), p=0.077, FDR=0.632 | -0.109 (0.043), p=0.012, FDR=0.202 | -0.063 (0.041), p=0.119, FDR=0.730 |
| PTau181 | 0.022 (0.039), p=0.573, FDR=0.941 | -0.023 (0.039), p=0.551, FDR=0.941 | 0.009 (0.039), p=0.814, FDR=0.944 | -0.068 (0.038), p=0.074, FDR=0.632 | -0.035 (0.039), p=0.363, FDR=0.865 | -0.016 (0.041), p=0.694, FDR=0.944 | -0.028 (0.038), p=0.455, FDR=0.865 |
| NfL | 0.084 (0.043), p=0.053, FDR=0.632 | -0.004 (0.043), p=0.924, FDR=0.944 | 0.020 (0.042), p=0.637, FDR=0.944 | 0.011 (0.041), p=0.791, FDR=0.944 | 0.049 (0.043), p=0.254, FDR=0.865 | -0.037 (0.045), p=0.403, FDR=0.865 | 0.056 (0.042), p=0.183, FDR=0.865 |
| GFAP | 0.128 (0.044), p=0.003, FDR=0.161 | 0.010 (0.044), p=0.824, FDR=0.944 | -0.007 (0.043), p=0.867, FDR=0.944 | -0.032 (0.043), p=0.459, FDR=0.865 | -0.041 (0.044), p=0.352, FDR=0.865 | -0.074 (0.046), p=0.108, FDR=0.730 | -0.053 (0.043), p=0.216, FDR=0.865 |
| Aβ42/40 ratio | -0.042 (0.049), p=0.385, FDR=0.865 | 0.012 (0.050), p=0.811, FDR=0.944 | -0.007 (0.048), p=0.889, FDR=0.944 | 0.046 (0.047), p=0.328, FDR=0.865 | -0.010 (0.048), p=0.841, FDR=0.944 | 0.040 (0.050), p=0.426, FDR=0.865 | 0.058 (0.049), p=0.235, FDR=0.865 |
| Aβ42 | 0.009 (0.049), p=0.858, FDR=0.944 | 0.064 (0.050), p=0.200, FDR=0.865 | -0.003 (0.048), p=0.958, FDR=0.958 | 0.049 (0.047), p=0.305, FDR=0.865 | 0.014 (0.048), p=0.771, FDR=0.944 | 0.055 (0.050), p=0.273, FDR=0.865 | 0.062 (0.049), p=0.201, FDR=0.865 |
| Aβ40 | 0.005 (0.044), p=0.919, FDR=0.944 | 0.022 (0.044), p=0.608, FDR=0.941 | -0.036 (0.043), p=0.414, FDR=0.865 | -0.007 (0.043), p=0.872, FDR=0.944 | -0.006 (0.044), p=0.896, FDR=0.944 | 0.029 (0.046), p=0.529, FDR=0.941 | -0.022 (0.043), p=0.607, FDR=0.941 |

Note: Neuro markers logged and z scored. All models adjusted for age, sex, education, rural residency, caste, smoking status, high blood pressure, high HbA1c, total to HDL cholesterol ratio, overweight, logged eGFR and depression, and for change models, the standardized baseline biomarker level at wave 1.

Supplementary Figure 1. Sample Selection
